# Soil Physical Properties Affect Herbivory of *Lampronadata cristata* in a Cork Oak Forest

**DOI:** 10.1002/ece3.70613

**Published:** 2024-11-18

**Authors:** Xinliang Shao, Xinjuan Zhou, Lin Wang, Ruxue Tan, Can Lu, Qin Zhang, Kedong Xu

**Affiliations:** ^1^ Key Laboratory of Plant Genetics and Molecular Breeding Zhoukou Normal University Zhoukou People's Republic of China; ^2^ College of Life Sciences and Agronomy Zhoukou Normal University Zhoukou People's Republic of China

**Keywords:** Cork oak, insect herbivory, leaf trait, soil nutrient, soil property

## Abstract

Studies have reported the important role of soil properties in regulating insect herbivory under controlled conditions or at relatively large scales. However, whether fine‐scale variation of soil properties affects insect herbivory under natural conditions in forests is still unclear. We selected a *ca.* 300 ha *Quercus variabilis* forest area where the leaf damage was mainly caused by *Lampronadata cristata* (Lepidoptera: Notodontidae) and set 200 10 × 10 m plots within the area. We examined insect herbivory (percent leaf area damaged) on *Q. variabilis* and correlated it to soil properties and tree characteristics. Insect herbivory decreased with soil sand percentage and bulk density and increased with DBH and tree height. Effects of soil sand percentage and bulk density on insect herbivory were partly mediated by DBH and tree height. Our results indicated that soil physical properties may have significant effects on insect herbivory by directly influencing insect herbivores that need to complete their life cycle in the soil, or by indirectly affecting insect herbivores through influencing DBH and tree height which reflects the total leaf biomass available to the insect herbivore. This study may help to understand the complex relationship between soil and plant–insect interactions in forest ecosystems.

## Graphical abstract

We examined the effects of soil properties and tree characteristics on herbivory caused by yellow two‐spotted prominent moth (lepidopterous insect) in a cork oak forest. Soil sand percentage and bulk density had significant negative effects on insect herbivory, and the effects were partly mediated by tree characteristics (DBH and tree height). The results indicated that soil physical properties may directly influence insect herbivores that need to spend part of their life in the soil, or indirectly affect insect herbivores through influencing the growth of focal host trees.

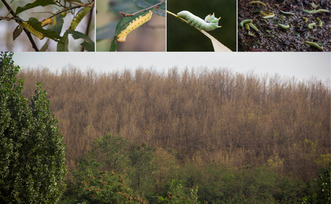

## 1 Introduction

Soil properties not only influence the growth, productivity, and reproductive success of plants (Van der Putten et al. 2013) but also impact the survival of insect herbivores that complete part of their life stages in the soil (Wainhouse 2005; Shao, Zhang, and Yang 2021). It is increasingly acknowledged that soil properties may play an important role in regulating insect herbivory (Schmitt and Burghardt 2021). The effects of soil properties on insect herbivory have been examined and reported in many studies. For example, nitrogen (N), phosphorus (P), and potassium (K) addition in a tropical forest increased both leaf N, P, and K concentrations and herbivory of five tropical tree seedling species (Santiago et al. 2012). Many other plant species have also been reported to suffered more leaf consumption when growing in environments with higher soil N (Bala et al. 2018). When compared between rural and urban areas, soil compaction has been recognized as an important factor influencing insect herbivory on trees. The compacted soil and ground hardening in urban areas may impede insect herbivores from digging into the soil for pupation or oviposition (Schmitt and Burghardt 2021), which could significantly increase the mortality of insect herbivores (Wen et al. 2016).

Soil moisture may also influence insect herbivory by directly and indirectly impacting the insect herbivore. Laboratory experiments demonstrated that both deficient (0%) and very high (80%) soil moisture significantly reduced the ability of *Heortia vitessoides* (Lepidoptera: Crambidae) to dig into the soil and thus increased their mortality (Wen et al. 2016). In the subtropical forest, soil moisture had significant indirect effects on insect herbivory by affecting plant growth and density (Li et al. 2021). In addition, studies have shown that variations in soil texture (percentage of clay, silt, and sand) have significant effects on insect herbivory (Shao, Zhang, and Yang 2021; Leite et al. 2022). Insect herbivory in gravel soil areas was significantly lower than in adjacent loam areas within *Quercus variabilis* forest stands (Shao, Zhang, and Yang 2021). The reason may be that gravel soil is more compact than loam and can reduce the number of mature larvae that successfully burrowed into the soil for pupating and overwintering (Shao, Zhang, and Yang 2021).

The examples demonstrated that soil chemical and physical properties have significant effects on insect herbivory by affecting host plant physiology and leaf palatability (Johnson et al. 2021; Massad et al. 2022; Bayissa et al. 2023) or by directly influencing the mortality of insect herbivores which spend part of their life in the soil (Wen et al. 2017; Shao, Zhang, and Yang 2021). However, most of the studies were carried out under controlled conditions (e.g., Santiago et al. 2012) or at relatively large scales such as comparisons between distant sites (Lynn and Fridley 2019). Whether fine‐scale variation of soil chemical and physical properties in forests under natural conditions has significant effects on insect herbivory is still unclear. Especially for herbivory caused by the lepidopterous defoliator which is one of the keystone pests in forests and many of them need to pupate and overwinter in the soil (Feeny 1970; Wainhouse 2005; Ji et al. 2011).

In this study, we examined insect herbivory within a *Q. variabilis* forest landscape where the herbivory (percent leaf area damaged) was primarily caused by *Lampronadata cristata* and correlated it to soil chemical and physical properties (e.g., soil available N, P, K, soil bulk density, soil texture, and soil moisture). Since host tree characteristics (e.g., stand density, tree height, and specific leaf area [SLA]) may also be influenced by soil properties and affect insect herbivory, we measured these factors and checked whether they mediate the effects of soil properties on insect herbivory. By addressing the effects of soil properties on insect herbivory, our study pursues a better understanding of the driving factors of insect herbivory in forests under natural conditions.

## 2 Materials and Methods

### 2.1 Study Area

This study was conducted in a *Q. variabilis* forest plantation in Lushan County, Henan Province, China (33°40′–33°42′ N, 112°46′–112°49′ E). The study area belongs to a warm temperate continental monsoon climate, the average annual temperature is 15.5°C, and the average annual precipitation is 825.6 mm. The soil types are mainly cinnamon soil and brown soil. This region is mainly covered by the cork oak ( *Q. variabilis* ) plantation (25–30 years old), with some scattered villages and agricultural lands in the remaining landscape. There are also a few other tree species (e.g., *Q. acutissima* , *Q. dentata* , *Q. aliena*) with minor abundance in the study area.

**FIGURE 1:**
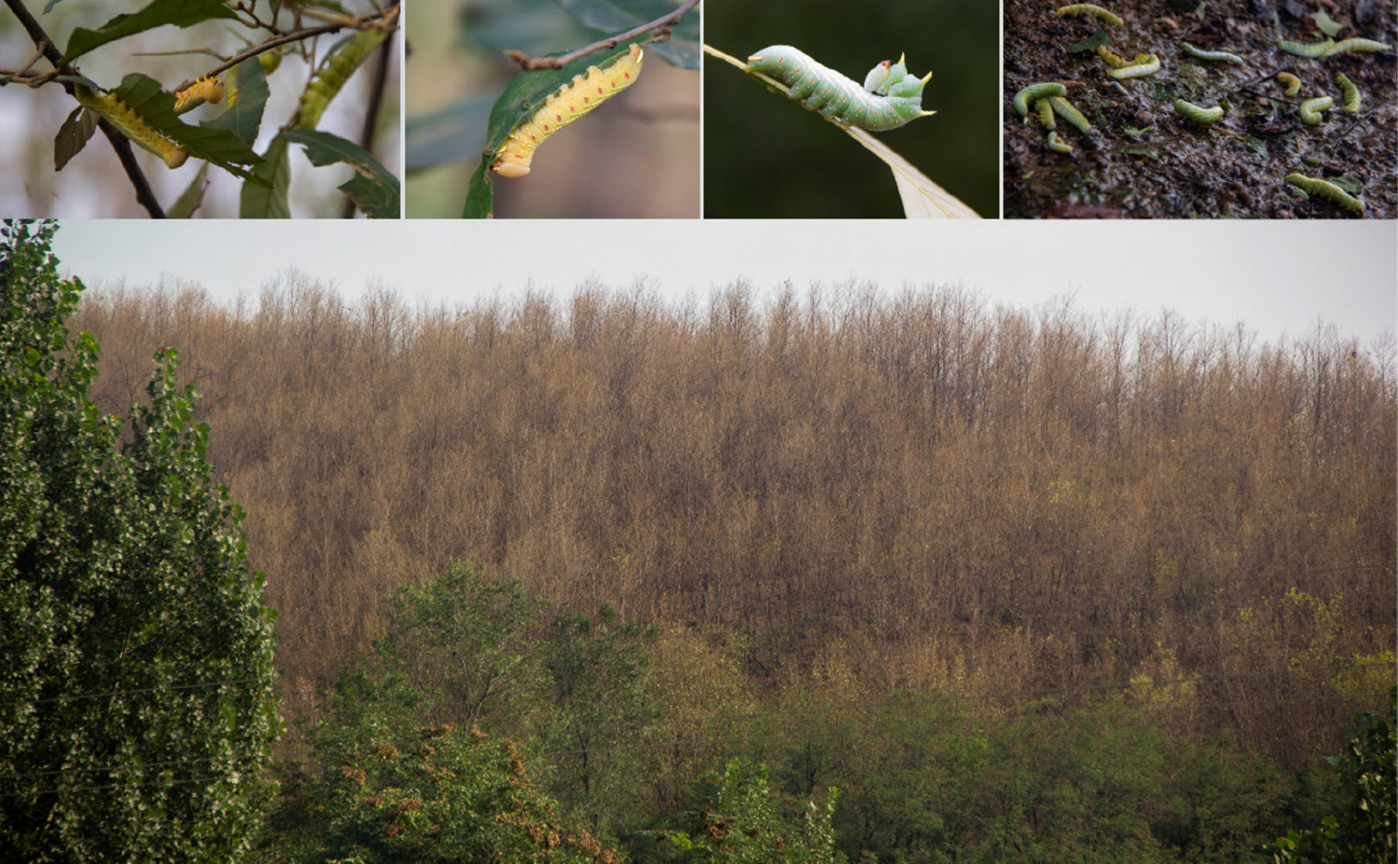
Photographs of *Lampronadata cristata* larvae and the severe defoliation of *Quercus variabilis* caused by this herbivore in the study area in August 2019.

The main insect herbivore on cork oak is *Lampronadata cristata* (Figure 1). *L. cristata* has two generations per year in this area. Adults begin to emerge in late May and the mature larvae dig into the soil and pupate in late July and early August. A part of the pupae enters the second generation and their mature larvae burrow into the soil for overwintering in mid‐October. There was an outbreak of this herbivore in 2019, and insect herbivory has remained at the background level since then.

We selected a *ca*. 300 ha area with less elevation variation (180–270 m) based on ground survey and set 200 10 × 10 m plots within the area and the distance between every two plots was at least 100 m.

### 2.2 Data Collection

Field investigation and sample collection were taken from late July to mid‐August 2022.

#### 2.2.1 Insect Herbivory

Three oak trees were randomly selected in each plot for leaf sampling. For each tree, mature leaves were randomly collected using a telescopic pole pruner, 15 from the top half and 15 from the bottom half of the crown. Thus, 30 leaves per individual tree and 90 leaves in each plot were collected. The leaves were put in plastic zip‐lock bags and stored in a cool box before being taken to the laboratory. For the measurement of insect herbivory, since nearly 90% of the insect larvae observed in the survey was *L. cristata* and other feeding guilds (e.g., skeletonizers and leaf‐miners) caused very little damage (below 5%), only the typical damages caused by chewers were estimated. The total leaf area and leaf missing area were calculated using the ImageJ software (Sam et al. 2020). Insect herbivory was calculated as the leaf missing area divided by the total leaf area and multiplied by 100%.

#### 2.2.2 Soil Properties

In each plot, soil moisture was measured using the soil moisture detector (SH‐TSJ, Shandong Shunhe Electronic Technology Co. Ltd.,) at three randomly selected points. We removed the litter until the decomposed leaves and branches cannot be recognized and collected soil using a 10 cm depth soil probe at these three points, mixed them together, and stored them as one sample in a plastic zip‐lock bag. The 10 cm depth may correspond to the humus horizon of soil. However, since the thick of humus horizon soil may be different among plots, the top 10 cm may also include eluvial horizon soil in some plots where the humus horizon is thin and the thick is below 10 cm. We avoided rainy days during the investigation and did not start collecting data until at least 24 h after the rain had stopped. Soil samples were air‐dried in the laboratory for about 1 month; then, they were sieved using a 2‐mm mesh. Soil total carbon and total nitrogen contents were determined using an elemental analyzer (Euro EA 3000; HEKAtech GmbH, Wegberg, Germany). Soil organic carbon, available nitrogen, available phosphorus, and available potassium contents were measured according to (Bao 2000). Mastersizer 2000 Particle Analyzer (Malvern Panalytical Ltd. Malvern, UK) was used to examine soil particle size. Soil bulk density was measured according to (Han et al. 2016).

#### 2.2.3 Tree Characteristics

In each plot, stand density, tree height, and DBH of *Q. variabilis* trees above 2 m were measured. For specific leaf area (SLA) and leaf water content measurement, 10 undamaged and fully expanded mature sun leaves were used in each plot. Leaf surface area was measured using a planimeter (CL‐203 Laser Area Meter; Bio‐Science Inc. USA). The leaves were weighed with a balance (TLE204E Electronic Balance, Mettler Toledo Instruments (Shanghai) Co. Ltd., China) before and after the 48 h oven‐dried at 60°C. SLA was calculated using leaf surface area divided by leaf dry weight, and leaf water content was measured as the leaf weight loss after drying divided by leaf fresh weight.

#### 2.2.4 Overwintering Experiment

In order to know whether *L. cristata* can successfully overwinter without digging into the soil, we collected approximately 150 mature larvae on August 20, 2022. The larvae were put into a plastic box (30 cm length × 15 cm width × 8 cm height) within an insect‐rearing cage (40 × 40 × 40 cm) in a room near the study area at ambient temperature and humidity. Almost all of the mature larvae pupated and about 30% of the pupae emerged as adult moths and entered the second generation. Then, 90 pupae were randomly selected and divided into three groups on October 16, 2022. Each group contained 30 pupae and was put into a mesh bag fastened on a 10 cm high platform within an insect‐rearing cage. The upper side of the cages was covered by plastic cloth for waterproofing. Cages were fixed to the forest floor with nails, and the distance between every two cages was about 50 m. The survival rate of pupae was examined on May 31, 2023.

#### 2.2.5 Statistical Analyses

The relationship between insect herbivory and environmental factors was determined by step‐wise regression analysis. We first examined the effects of soil properties (soil C, N, organic carbon, available N, P, K, soil moisture, soil bulk density, percentage of sand) on insect herbivory. Since host tree characteristics (stand density, diameter at breast height (DBH), tree height, SLA, and leaf water content) may also affect insect herbivory and be influenced by soil properties, we were interested in distinguishing the effects of soil properties and host tree characteristics on insect herbivory. We examined the effects of host tree characteristics on insect herbivory in a separate model. Based on the results of step‐wise regression analyses, we then used structural equation modeling (SEM) to investigate the direct and indirect effects of the main influencing factors (percentage of soil sand, soil bulk density, DBH, and tree height) on insect herbivory. The percentage of soil sand and soil bulk density were exogeneous variables, and DBH and tree height were endogeneous variables. We initially hypothesized all the paths in the network contain useful information and compared the fitness among all the possible models. The model was considered rejected if the *p* value of *χ* ^2^ test of the model fell below the significance level (*p* < 0.05) and the best model was retained according to (Purwanto, Fahmi, and Sulaiman 2023). Linear regression analysis was used to examine the correlation between insect herbivory and each of the main influencing factors.

Step‐wise regression analyses were conducted using IBM SPSS Statistics 20 (SPSS Inc. Chicago, IL, USA), and scatter diagrams were plotted using Origin 2018 (OriginLab; Northampton, MA, USA). The SEM was performed using IBM SPSS Amos 26 (SPSS Inc. Chicago, IL, USA).

## 3 Results

**FIGURE 2:**
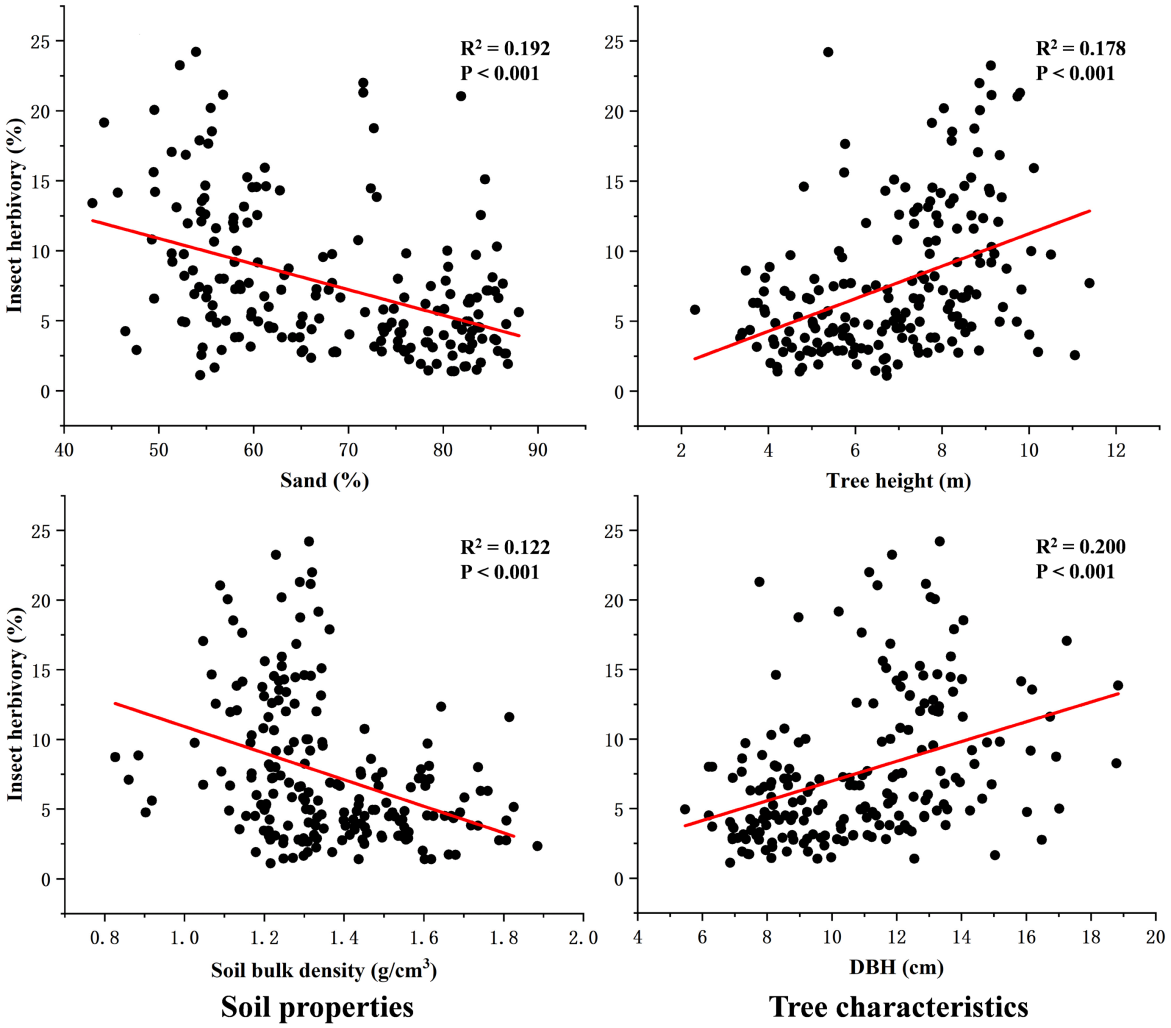
Effects of soil sand percentage, soil bulk density, tree height, and DBH on insect herbivory. Dots show the original data.

Descriptive statistics for the variables (insect herbivory, soil properties, and tree characteristics) were summarized in Table 1. The average herbivory (percent leaf area damaged) (± SE) was 7.53% ± 0.36%, corresponding to the background level of insect herbivory in this study area. Step‐wise regression analysis between soil properties and insect herbivory retained two predictive variables (percentage of sand and soil bulk density) in the optimal model (Table 2). Insect herbivory was negatively correlated with soil sand percentage and bulk density (Table 2, Figure 2). The predictive variables DBH and tree height were retained in the optimal model of step‐wise regression analysis between host tree characteristics and insect herbivory (Table 2). Insect herbivory was positively correlated with DBH and tree height (Table 2, Figure 2). Soil nutrients (soil C, N, organic carbon, available N, P, K, and soil moisture) and leaf traits (SLA and leaf water content) have no significant effects on insect herbivory measured as percent area damaged. In the pupa survival experiment, not a single pupa survived until late May 2023, indicating that *L. cristata* cannot successfully overwinter without digging into the soil.

**TABLE 1:** Descriptive statistical results of the variables (insect herbivory, soil properties and tree characteristics) examined in the experiment.

Variables	Sample size	Minimum	Maximum	Average	Standard error
Insect herbivory (% leaf area)	200	1.10	24.20	7.53	0.36
Soil N (mg/kg)	200	0.81	9.50	2.15	0.07
Soil C (mg/kg)	200	9.17	105.13	24.71	0.79
Soil organic C (mg/kg)	200	7.01	80.42	18.90	0.59
Available N (mg/kg)	200	12.00	470.03	56.68	3.62
Available P (mg/kg)	200	0.92	1.82	1.12	0.01
Available K (mg/kg)	200	51.15	475.89	125.03	4.25
Soil moisture (%)	200	4.98	17.69	11.45	0.21
Soil bulk density (g/cm^3^)	200	0.83	1.88	1.36	0.01
Sand (%)	200	42.99	87.98	68.31	0.86
Stand density (plants/ha)	200	600	2700	1430.50	20.28
Tree height (m)	200	2.31	11.39	6.80	0.13
DBH (cm)	200	5.46	18.84	10.79	0.19
SLA (m^2^/kg)	200	7.77	14.65	10.57	0.09
Leaf water content (%)	200	0.37	0.52	0.45	0.002

**TABLE 2:** Results of step‐wise regression analyses between soil properties/host tree characteristics and insect herbivory.

Soil properties‐herbivory *R* ^2^ = 0.236, *F*_(2, 197)_ = 30.475	Host tree characteristics‐herbivory *R* ^2^ = 0.228, *F*_(2, 197)_ = 29.167
Sand	DBH
*t* = −6.853, *p* < 0.001, VIF = 1.135	*t* = 3.598, *p* < 0.001, VIF = 1.760
Soil bulk density	Tree height
*t* = −3.391, *p* = 0.001, VIF = 1.135	*t* = 2.715, *p* = 0.007, VIF = 1.760

**FIGURE 3:**
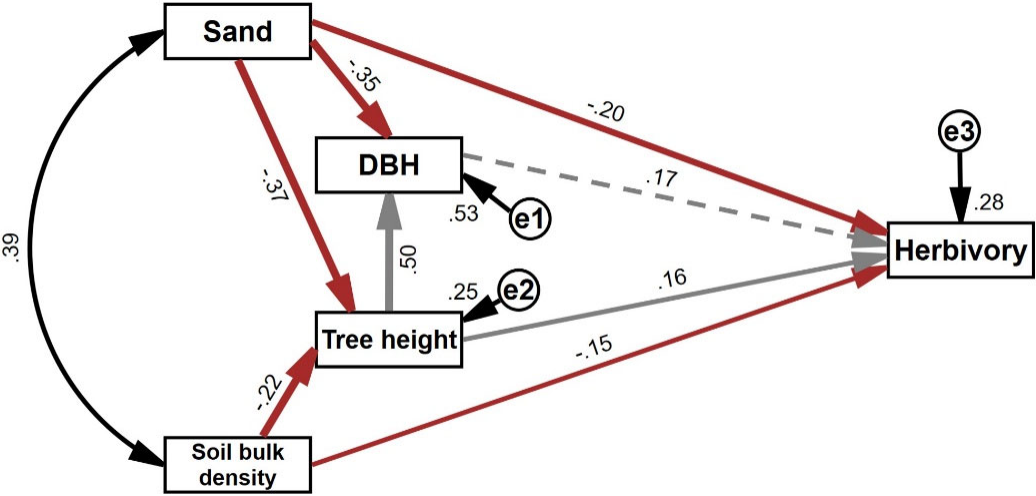
Path diagrams showing direct and indirect effects on insect herbivory. Solid brown and gray lines represent significant negative and positive relationships, respectively, and the line width corresponds to the significant value of the analysis, with more significant values represented by wider lines. The dashed gray line represents a non‐significant positive relationship. Arrows indicate the direction of effects. Standard coefficients are shown along paths. The e1, e1, and e3 are errors of observed variable.

In order to ascertain whether the effects of soil properties on insect herbivory were mediated by host plant characteristics, we examined the direct and indirect effects of soil sand percentage, bulk density, DBH, and tree height on insect herbivory using SEM. Results of SEM (*χ* ^2^ = 1.06, *p* = 0.30) confirmed that soil sand percentage and bulk density have significant direct and indirect effects on insect herbivory, and the indirect effects were mediated by DBH and tree height (Figure 3).

## 4 Discussion

Our study shows that higher soil sand percentage and bulk density reduced insect herbivory measured as percent leaf area damaged and the effects were partly mediated by DBH and tree height (Table 2, Figures 2 and 3). The soil not only provides nutrients for plants but also provides habitats (including feeding, pupating, and overwintering) for insect herbivores. Thus, soil may interact directly with insect herbivores and influence their feeding behavior and mortality. Soil sand percentage and soil bulk density may reflect soil compaction, and high soil compaction can negatively influence insect herbivory by affecting the ability of insect herbivores to dig into the soil for pupation (Wen et al. 2016; Schmitt and Burghardt 2021). That may explain why insect herbivory negatively correlated with soil sand percentage and bulk density.

Previous studies have reported that insect herbivory was positively correlated with DBH of *Q. castanea* and *Q. obtusata* (Pérez‐Solache et al. 2023) and with the basal area of *Pinus pinaster* (Poeydebat et al. 2021), four other evergreen tree species (Martini, Sun, and Chen 2022) and 19 savanna tree species (Martini et al. 2021). Our results are consistent with these studies (Table 2, Figures 2 and 3). DBH and tree height promoted insect herbivory, likely because they reflect the total amount of resources available to insect herbivores (Poeydebat et al. 2021). Higher resource concentration possibly attracts more insect herbivores through increased visual and olfactory apparency of host trees (Castagneyrol et al. 2013, 2019; Martini et al. 2021). Martini et al. (2021) also reported that plant apparency was the main driver of insect herbivory in subtropical forests. Even in pure forest, previous research has shown that taller pines ( *Pinus pinaster* ) within stands are more susceptible to infestation by the pine processionary moth (*Thaumetopoea pityocampa*) (Régolini et al. 2014). Beyond direct effects, there were also indirect effects of soil sand percentage and bulk density on insect herbivory by negatively influencing DBH and tree height (Figure 3). This result is in line with previous studies which have reported that soil with higher sand percentage and bulk density can negatively influence plant root growth (Jalota et al. 2010; Tracy et al. 2013), implying that soil physical properties play an important role in regulating plant growth and thus affect insect herbivory.

Leaf traits measured in our study did not have significant effects on insect herbivory. This result is consistent with our previous study (Shao et al. 2024), suggesting once again that variation in leaf traits of the same host tree species may not have significant effects on insect herbivory in similar environments, that is, the effects of leaf traits might be masked by other factors (Shao et al. 2024). As for the results that soil nutrients did not significantly influence insect herbivory, this is inconsistent with many previous studies conducted through nutrient manipulation under controlled conditions (Richardson et al. 2002; Prudic, Oliver, and Bowers 2005; Santiago et al. 2012), but it is consistent with the study that reported soil N, P, and K content had no significant effects on insect herbivory in the understory seedling community of subtropical forests under natural conditions (Martini et al. 2021). This suggests that the effects of soil nutrients on insect herbivory may vary greatly under controlled and natural conditions. Researchers should be cautious when applying results from controlled conditions to natural environments, where there are inevitably many additional factors that may influence the absorption and utilization of nutrients by plants and thus affect insect herbivory.

For instance, it is well known that roots of almost all land plants (about 80%–90%) are capable of forming mycorrhiza with fungi (Wang and Qiu 2006; Brundrett 2009). About 80% of nitrogen and phosphorus is supplied to plants by mycorrhizal fungi (van der Heijden et al. 2015). The mycelium of plant mycorrhiza, through extension and fusion, can form a huge underground network connecting various plants (Fortuna and Pellegrino 2006; Simard 2009; van der Heijden et al. 2015; Genre et al. 2020), and different plants can transmit signals and nutrients to each other through the mycelium network (Fortuna and Pellegrino 2006; Simard 2009). Previous studies have reported that the growth of *Q. variabilis* can be promoted by forming symbionts with mycorrhizal fungi (Wang et al. 2019; Wei, Song, and Jia 2020; Huang et al. 2022). Thus, we speculate that soil nutrients in our study area did not have significant effects on insect herbivory might be related to mycorrhizal fungi that form symbionts with *Q. variabilis* . This may be very different from that under controlled conditions where different plant individuals lack nutrient delivery through mycorrhizal mycelium network or the role of mycorrhizal fungi was masked by the overwhelming effects of soil nutrient manipulation (e.g., Santiago et al. 2012; Johnson et al. 2021). Some studies have reported the significant effects of mycorrhizal fungi on insect herbivory. For example, mycorrhizal fungi can affect the growth and development of *Acyrthosiphon pisum* and *Spodoptera exigua* by changing the quality of host plants or influence the recruitment of insect herbivores by altering the release of host plant volatiles, thus having significant effects on insect herbivory (Babikova et al. 2014; Wang et al. 2015). Therefore, we can further speculate that there may be complex mycorrhizal networks among the roots of different oak individuals in the study area, and nutrient elements can be transmitted among different individuals, so that the variation of soil nutrient status does not significantly affect plant growth and insect herbivory. However, further experiments are needed to confirm this.

In conclusion, our study highlights the significant effects of soil physical properties on insect herbivory of *L. cristata* in the *Q. variabilis* forest and suggests that the effects of soil nutrients and leaf traits on insect herbivory under natural conditions might be very different from that under controlled conditions. These findings may help to understand the complex driving forces of forest insect herbivory in real‐world contexts and have implications for sustainable forest management.

## Author Contributions

**Xinliang Shao:** conceptualization (equal), data curation (equal), formal analysis (equal), funding acquisition (equal), investigation (equal), methodology (equal), project administration (equal), resources (equal), software (equal), supervision (equal), validation (equal), visualization (equal), writing – original draft (equal), writing – review and editing (equal). **Xinjuan Zhou:** investigation (equal), methodology (equal), writing – original draft (equal), writing – review and editing (equal). **Lin Wang:** investigation (equal), methodology (equal), writing – original draft (equal), writing – review and editing (equal). **Ruxue Tan:** investigation (equal), methodology (equal), writing – original draft (equal), writing – review and editing (equal). **Can Lu:** investigation (equal), methodology (equal), writing – original draft (equal), writing – review and editing (equal). **Qin Zhang:** conceptualization (equal), data curation (equal), formal analysis (equal), funding acquisition (equal), investigation (equal), methodology (equal), project administration (equal), resources (equal), software (equal), supervision (equal), validation (equal), visualization (equal), writing – original draft (equal), writing – review and editing (equal). **Kedong Xu:** conceptualization (equal), data curation (equal), formal analysis (equal), funding acquisition (equal), investigation (equal), methodology (equal), project administration (equal), resources (equal), supervision (equal), writing – original draft (equal), writing – review and editing (equal).

## Conflicts of Interest

The authors declare no conflicts of interest.

## Supporting information


Data S1.

## Data Availability

Raw data used in this work are provided in Supporting Information.

## References

[ece370613-bib-0001] Babikova, Z. , L. Gilbert , T. Bruce , S. Y. Dewhirst , J. A. Pickett , and D. Johnson . 2014. “Arbuscular Mycorrhizal Fungi and Aphids Interact by Changing Host Plant Quality and Volatile Emission.” Functional Ecology 28: 375–385.

[ece370613-bib-0002] Bala, K. , A. Sood , V. S. Pathania , and S. Thakur . 2018. “Effect of Plant Nutrition in Insect Pest Management: A Review.” Journal of Pharmacognosy and Phytochemistry 7: 2737–2742.

[ece370613-bib-0003] Bao, S. 2000. Soil Agrochemical Analysis. Beijing: China Agricultural Press. (in Chinese).

[ece370613-bib-0004] Bayissa, W. , A. Abera , J. Temesgen , G. Abera , and E. Mendesil . 2023. “Organic Soil Fertility Management Practices for the Management of Fall Armyworm, *Spodoptera frugiperda* (JE Smith), in Maize.” Frontiers in Insect Science 3: 1210719. 38469541 10.3389/finsc.2023.1210719 PMC10926535

[ece370613-bib-0005] Brundrett, M. C. 2009. “Mycorrhizal Associations and Other Means of Nutrition of Vascular Plants: Understanding the Global Diversity of Host Plants by Resolving Conflicting Information and Developing Reliable Means of Diagnosis.” Plant and Soil 320: 37–77.

[ece370613-bib-0006] Castagneyrol, B. , B. Giffard , C. Péré , and H. Jactel . 2013. “Plant Apparency, an Overlooked Driver of Associational Resistance to Insect Herbivory.” Journal of Ecology 101: 418–429.

[ece370613-bib-0007] Castagneyrol, B. , B. Giffard , E. Valdés‐Correcher , and A. Hampe . 2019. “Tree Diversity Effects on Leaf Insect Damage on Pedunculate Oak: The Role of Landscape Context and Forest Stratum.” Forest Ecology and Management 433: 287–294.

[ece370613-bib-0008] Feeny, P. 1970. “Seasonal Changes in Oak Leaf Tannins and Nutrients as a Cause of Spring Feeding by Winter Moth Caterpillars.” Ecology 51: 565–581.

[ece370613-bib-0009] Fortuna, P. , and E. Pellegrino . 2006. “Review at the Root of the Wood Wide Web.” Plant Signaling & Behavior 1: 1–5. 19521468 10.4161/psb.1.1.2277 PMC2633692

[ece370613-bib-0010] Genre, A. , L. Lanfranco , S. Perotto , and P. Bonfante . 2020. “Unique and Common Traits in Mycorrhizal Symbioses.” Nature Reviews. Microbiology 18: 649–660. 32694620 10.1038/s41579-020-0402-3

[ece370613-bib-0011] Han, Y. , J. Zhang , K. G. Mattson , W. Zhang , and T. A. Weber . 2016. “Sample Sizes to Control Error Estimates in Determining Soil Bulk Density in California Forest Soils.” Soil Science Society of America Journal 80: 756–764.

[ece370613-bib-0012] Huang, L.‐L. , Y.‐L. Wang , A. Guerin‐Laguette , et al. 2022. “Ectomycorrhizal Synthesis Between Two Tuber Species and Six Tree Species: Are Different Host‐Fungus Combinations Having Dissimilar Impacts on Host Plant Growth?” Mycorrhiza 32: 341–351. 35608677 10.1007/s00572-022-01081-6

[ece370613-bib-0013] Jalota, S. , S. Singh , G. Chahal , S. Ray , S. Panigraghy , and K. Singh . 2010. “Soil Texture, Climate and Management Effects on Plant Growth, Grain Yield and Water Use by Rainfed Maize–Wheat Cropping System: Field and Simulation Study.” Agricultural Water Management 97: 83–90.

[ece370613-bib-0014] Ji, L. , Z. Wang , X. Wang , and L. An . 2011. “Forest Insect Pest Management and Forest Management in China: An Overview.” Environmental Management 48: 1107–1121. 21667316 10.1007/s00267-011-9697-1

[ece370613-bib-0015] Johnson, S. N. , J. M. Waterman , R. Wuhrer , R. C. Rowe , C. R. Hall , and X. Cibils‐Stewart . 2021. “Siliceous and Non‐Nutritious: Nitrogen Limitation Increases Anti‐Herbivore Silicon Defences in a Model Grass.” Journal of Ecology 109: 3767–3778.

[ece370613-bib-0016] Leite, G. L. D. , R. Veloso , M. A. Soares , et al. 2022. “Changes in Galling Insect Community on *Caryocar brasiliense* Trees Mediated by Soil Chemical and Physical Attributes.” Brazilian Journal of Biology 82: e261227. 10.1590/1519-6984.261227 35976355

[ece370613-bib-0017] Li, W. , Y. Chen , Y. Shen , Y. Lu , and S. Yu . 2021. “Plant Trait Differences and Soil Moisture Jointly Affect Insect Herbivory on Seedling Young Leaves in a Subtropical Forest.” Forest Ecology and Management 482: 118878.

[ece370613-bib-0018] Lynn, J. , and J. Fridley . 2019. “Geographic Patterns of Plant—Herbivore Interactions Are Driven by Soil Fertility.” Journal of Plant Ecology 12: 653–661.

[ece370613-bib-0019] Martini, F. , S. T. Aluthwattha , C. Mammides , M. Armani , and U. M. Goodale . 2021. “Plant Apparency Drives Leaf Herbivory in Seedling Communities Across Four Subtropical Forests.” Oecologia 195: 575–587. 33251556 10.1007/s00442-020-04804-8

[ece370613-bib-0020] Martini, F. , I.‐F. Sun , and Y.‐Y. Chen . 2022. “Effects of Plant Diversity and Leaf Traits on Insect Herbivory in Plantation and Natural Forests.” Forest Ecology and Management 509: 120085.

[ece370613-bib-0021] Martini, V. C. , D. Raymundo , J. Prado‐Junior , and D. C. Oliveira . 2021. “Bottom‐Up and Top‐Down Forces in Plant‐Gall Relationships: Testing the Hypotheses of Resource Concentration, Associational Resistance, and Host Fitness Reduction.” Ecological Entomology 46: 1072–1081.

[ece370613-bib-0022] Massad, T. J. , L. A. Richards , C. Philbin , et al. 2022. “The Chemical Ecology of Tropical Forest Diversity: Environmental Variation, Chemical Similarity, Herbivory, and Richness.” Ecology 103: e3762. 35593436 10.1002/ecy.3762

[ece370613-bib-0023] Pérez‐Solache, A. , M. S. Vaca‐Sánchez , Y. Maldonado‐López , et al. 2023. “Changes in Land Use of Temperate Forests Associated to Avocado Production in Mexico: Impacts on Soil Properties, Plant Traits and Insect‐Plant Interactions.” Agricultural Systems 204: 103556.

[ece370613-bib-0024] Poeydebat, C. , B. Castagneyrol , I. Van Halder , and H. Jactel . 2021. “Changes in Host Basal Area Explain Associational Resistance of Mixed Forests to Primary Pests.” Forest Ecology and Management 495: 119374.

[ece370613-bib-0025] Prudic, K. L. , J. C. Oliver , and M. D. Bowers . 2005. “Soil Nutrient Effects on Oviposition Preference, Larval Performance, and Chemical Defense of a Specialist Insect Herbivore.” Oecologia 143: 578–587. 15909129 10.1007/s00442-005-0008-5

[ece370613-bib-0026] Purwanto, A. , K. Fahmi , and A. Sulaiman . 2023. “Linking of Transformational Leadership, Learning Culture, Organizational Structure and School Innovation Capacity: CB SEM AMOS Analysis.” Journal of Information Systems Management 2: 1–8.

[ece370613-bib-0027] Régolini, M. , B. Castagneyrol , A.‐M. Dulaurent‐Mercadal , D. Piou , J.‐C. Samalens , and H. Jactel . 2014. “Effect of Host Tree Density and Apparency on the Probability of Attack by the Pine Processionary Moth.” Forest Ecology and Management 334: 185–192.

[ece370613-bib-0028] Richardson, S. J. , M. C. Press , A. N. Parsons , and S. E. Hartley . 2002. “How Do Nutrients and Warming Impact on Plant Communities and Their Insect Herbivores? A 9‐Year Study From a Sub‐Arctic Heath.” Journal of Ecology 90: 544–556.

[ece370613-bib-0029] Sam, K. , B. Koane , L. Sam , et al. 2020. “Insect Herbivory and Herbivores of *Ficus* Species Along a Rain Forest Elevational Gradient in Papua New Guinea.” Biotropica 52: 263–276.

[ece370613-bib-0030] Santiago, L. S. , S. J. Wright , K. E. Harms , et al. 2012. “Tropical Tree Seedling Growth Responses to Nitrogen, Phosphorus and Potassium Addition.” Journal of Ecology 100: 309–316.

[ece370613-bib-0031] Schmitt, L. , and K. T. Burghardt . 2021. “Urbanization as a Disrupter and Facilitator of Insect Herbivore Behaviors and Life Cycles.” Current Opinion in Insect Science 45: 97–105. 33676055 10.1016/j.cois.2021.02.016

[ece370613-bib-0032] Shao, X. , K. Cheng , Q. Zhang , F. Xu , and L. Li . 2024. “Do Leaf Traits Affect Insect Herbivory in a Chinese Cork Oak Forest?” New Zealand Journal of Forestry Science 54: 1–10.

[ece370613-bib-0033] Shao, X. , Q. Zhang , and X. Yang . 2021. “Spatial Patterns of Insect Herbivory Within a Forest Landscape: The Role of Soil Type and Forest Stratum.” Forest Ecosystems 8: 69.

[ece370613-bib-0034] Simard, S. W. 2009. “The Foundational Role of Mycorrhizal Networks in Self‐Organization of Interior Douglas‐Fir Forests.” Forest Ecology and Management 258: S95–S107.

[ece370613-bib-0035] Tracy, S. R. , C. R. Black , J. A. Roberts , and S. J. Mooney . 2013. “Exploring the Interacting Effect of Soil Texture and Bulk Density on Root System Development in Tomato ( *Solanum lycopersicum* L.).” Environmental and Experimental Botany 91: 38–47.

[ece370613-bib-0036] van der Heijden, M. G. A. , F. M. Martin , M.‐A. Selosse , and I. R. Sanders . 2015. “Mycorrhizal Ecology and Evolution: The Past, the Present, and the Future.” New Phytologist 205: 1406–1423. 25639293 10.1111/nph.13288

[ece370613-bib-0037] Van der Putten, W. H. , R. D. Bardgett , J. D. Bever , et al. 2013. “Plant‐Soil Feedbacks: The Past, the Present and Future Challenges.” Journal of Ecology 101: 265–276.

[ece370613-bib-0038] Wainhouse, D. 2005. Ecological Methods in Forest Pest Management. Oxford University Press, New York.

[ece370613-bib-0039] Wang, B. , and Y.‐L. Qiu . 2006. “Phylogenetic Distribution and Evolution of Mycorrhizas in Land Plants.” Mycorrhiza 16: 299–363. 16845554 10.1007/s00572-005-0033-6

[ece370613-bib-0040] Wang, M. , T. M. Bezemer , W. H. Van der Putten , and A. Biere . 2015. “Effects of the Timing of Herbivory on Plant Defense Induction and Insect Performance in Ribwort Plantain ( *Plantago lanceolata* L.) Depend on Plant Mycorrhizal Status.” Journal of Chemical Ecology 41: 1006–1017. 26552915 10.1007/s10886-015-0644-0 PMC4670619

[ece370613-bib-0041] Wang, R. , A. Guerin‐Laguette , R. Butler , L.‐L. Huang , and F.‐Q. Yu . 2019. “The European Delicacy *Tuber melanosporum* Forms Mycorrhizae With Some Indigenous Chinese *Quercus* Species and Promotes Growth of the Oak Seedlings.” Mycorrhiza 29: 649–661. 31760479 10.1007/s00572-019-00925-y

[ece370613-bib-0042] Wei, S. , Y. Song , and L. Jia . 2020. “Influence of the Slope Aspect on the Ectomycorrhizal Fungal Community of *Quercus variabilis* Blume in the Middle Part of the Taihang Mountains, North China.” Journal of Forestry Research 32: 385–400.

[ece370613-bib-0043] Wen, Y. , X. Jin , C. Zhu , et al. 2016. “Effect of Substrate Type and Moisture on Pupation and Emergence of *Heortia vitessoides* (Lepidoptera: Crambidae): Choice and No‐Choice Studies.” Journal of Insect Behavior 29: 473–489.

[ece370613-bib-0044] Wen, Y. , W. Qin , X. Chen , et al. 2017. “Soil Moisture Effects on Pupation Behavior, Physiology, and Morphology of *Heortia vitessoides* (Lepidoptera: Crambidae).” Journal of Entomological Science 52: 229–238.

